# End-on versus parallel radiofrequency lesioning for neurotomy of the cervical medial branch nerves: a study protocol of a prospective, randomized, double-blind clinical trial: the “EndPaRL” study

**DOI:** 10.1186/s13063-023-07752-9

**Published:** 2023-11-11

**Authors:** Abeer Alomari, Guilherme Ferreira-Dos-Santos, Mandeep Singh, Taylor Burnham, Xingshan Cao, Zachary McCormick, David Flamer, Pranab Kumar, Yasmine Hoydonckx, James S. Khan, Paul S. Tumber, Danielle Alvares, Anuj Bhatia

**Affiliations:** 1 Department of Anesthesia and Pain Medicine, University of Toronto, Toronto Western Hospital, McL 2-405, 399 Bathurst Street, Toronto, Ontario M5T 2S8 Canada; 2 Division of Pain Medicine, Department of Anesthesiology, Reanimation and Pain Medicine, Hospital Clínic de Barcelona, University of Barcelona, Calle de Villarroel, 170, 08036 Barcelona, Spain; 3 Department of Anesthesiology and Pain Medicine, University Health Network, Women’s College Hospital, University of Toronto, Toronto, ON Canada; 4 Department of Physical Medicine and Rehabilitation, Spine and Musculoskeletal Rehabilitation Section, University of Utah, 50 North Medical Drive, Salt Lake City, UT 84132 USA; 5 Research Design and Biostatistics, Sunnybrook Health Sciences Centre, Toronto, ON Canada; 6 Department of Anesthesia and Pain Medicine, University of Toronto, Sinai Health System, Toronto, ON Canada; 7 Institute of Health Policy, Management and Evaluation, University of Toronto, Toronto, ON Canada

**Keywords:** Neck pain, Cervical medial branch nerve, Radiofrequency ablation, Pain, Non-inferiority

## Abstract

**Background:**

Cervical facet joint disease is a common source of neck pain and its prevalence increases with aging. Conservative multimodal management options (e.g., strengthening of neck muscles, non-steroidal anti-inflammatory medications, massage, and thermal modalities) often fail to relieve pain. Cervical medial branch nerve (CMBN) radiofrequency neurotomy (RFN) is an effective minimally invasive technique for treating chronic neck pain secondary to facet joint disease. An end-on approach for this procedure has been proposed that may be technically easier and require less time while reducing post-procedural discomfort. The protocol presented here is for a study that aims to compare the efficacy of a new end-on approach using multi-tined cannulae, against the conventional parallel technique that employs straight cannulae for RFN of the CMBN in patients with chronic neck pain due to cervical facet joint disease.

**Methods:**

A multicentre randomized, non-inferior, active comparator-controlled trial will be conducted with two parallel groups and blinding of participants and outcome assessor. The study will include 72 adults with chronic neck pain secondary to facet joint disease who are candidates for RFA of the CMBN. Participants will be randomized to either the conventional parallel or the end-on approach in a 1:1 ratio. The intensity of pain and pain-related domains (function, quality of life, sleep, adverse effects of the interventions, analgesic intake) will be measured at 1, 3, 6, and 12 months after the procedure.

**Discussion:**

Neck pain secondary to cervical facet joint disease is prevalent and RFA of the CMBN is a validated treatment for relieving it. The conventional parallel technique can be technically challenging, and it can be associated with adverse effects while the newer end-on approach has the potential of being a simpler technique with less adverse effects. This trial will be the first non-inferiority study to compare the clinical efficacy of the end-on approach against the conventional parallel approach for RFN of CMBN in patients with chronic neck pain due to cervical facet joint disease.

**Trial registration:**

ClinicalTrials.gov NCT05818774. Registered on April 20, 2023.

**Supplementary Information:**

The online version contains supplementary material available at 10.1186/s13063-023-07752-9.

## Administrative information

World Health Organization Registration Data Set

**Table:** 

Title	End-on Versus Parallel Radiofrequency Lesioning for Neurotomy of the Cervical Medial Branch Nerves: a Study Protocol of a prospective, randomized, Double-Blind Clinical Trial: The “EndPaRL” Study
Primary registry and trial identifying number	ClinicalTrials.gov NCT05818774
Other protocol identifier	22-5634
Date of registration in primary registry	2023-04-20
Protocol version	Version 3.0 dated 27Jul2023
Funding	The Spine Intervention Society and the Department of Anesthesia and Pain Management, Toronto Western Hospital, University Health Network, 399 Bathurst Street, Toronto, Ontario, Canada
Author details	Abeer Alomari MBBS MD Mandeep Singh MBBS MD MSc FRCPC Pranab Kumar MD FRCA FRCPC LLM Paul S. Tumber MD, FRCPC Danielle Alvares PhD Yasmine Hoydonckx MD MSc FIPP Anuj Bhatia MBBS MD PhD FRCPC Department of Anesthesia and Pain Medicine, University of Toronto, Toronto Western Hospital, McL 2-405, 399 Bathurst Street, Toronto, Ontario, M5T 2S8, Canada Guilherme Ferreira-Dos-Santos MD Division of Pain Medicine, Department of Anesthesiology, Reanimation and Pain Medicine,Hospital Clínic de Barcelona, University of Barcelona. Calle de Villarroel, 170, 08036, Barcelona, Spain. Xingshan Cao PhD Research Design and Biostatistics, Sunnybrook Health Sciences Centre, Toronto, Ontario, Canada Zachary McCormick MD FAAPMR Taylor Burnham DO MSCI Department of Physical Medicine and Rehabilitation, Spine and Musculoskeletal Rehabilitation Section, University of Utah, 50 North Medical Drive, Salt Lake City, Utah 84132 David Flamer MD FRCPC James S. Khan, MSc MD FRCPC Department of Anesthesia and Pain Medicine, University of Toronto, Sinai Health System, Toronto, Ontario, Canada
Primary sponsor	Department of Anesthesia and Pain Management University Health Network 399 Bathurst Street, 2-McL Toronto, ON M5T 2S8 Tel: (416) 603-5800 ext. 6237 Fax: (416) 603-6494
Contact for public queries	Department of Anesthesia and Pain Management, Toronto Western Hospital, University Health Network, 399 Bathurst Street, Toronto, Ontario, Canada
Contact for scientific queries	Principal Investigator: Anuj Bhatia MD, PhD, FRCPC Toronto Western Hospital, University Health Network 399 Bathurst Street, 2-McL 405, Toronto, Ontario M5T 2S8 Anuj.Bhatia@uhn.ca Tel: (416) 603-5800 ext. 5118 Fax: (416) 603-6494 Contact for scientific queries: Kawalpreet Singh MD Trial Coordinator, Department of Anesthesia and Pain Management, Toronto Western Hospital, University Health Network, 399 Bathurst Street, 2-McL 405, Toronto, Ontario Canada M5T 2S8 kawal.singh@ uhn.ca Tel: (416) 603-5800 ext. 5118 Fax: (416) 603-6494
Role of sponsor	The sponsor or funders have no role in the trial design, collection,management, analysis, and interpretation of the data; writing of the report; and the decision to submit the report for publication.

## Introduction

### Background and rationale

Parallel radiofrequency neurotomy (RFN) of the cervical medial branch nerves (CMBN) utilizing sharp straight needles was first demonstrated to be clinically efficacious in a randomized clinical trial (RCT) setting for the treatment of chronic neck pain following whiplash injury almost three decades ago [1]. Over the next 20 years, several other studies followed with variable success rates. In the early 2000s, in a consecutive series of cases, the authors reported a success rate (defined as the complete abolition of pain) of 46 and 26% following RFN of the CMBN at 6 and 12 months, respectively [2]. Previously, an observational study in the late 1990s had already reported a success rate (defined as abolition of pain) of 57 and 36% at the 6- and 12-month follow-up, respectively [3]. Later, in 2012, a study conducted in New Zealand reported a success rate (defined as greater than 80% pain relief) of 68 and 51% at 6 and 12 months post-procedurally, respectively [4]. In addition, the rate of complications between studies also showed variability throughout the years. The rates of temporary post-procedural dysesthesias have been noted to vary from 43 to 67%, while the rates of temporary cutaneous numbness have been reported to range between 36 and 58%. Of note, only the rates of post-procedural pain seem to be relatively concordant between authors, with reported rates varying between 95 and 100% among studies. This data was comprehensively analyzed in a systematic review published in the late 2010s by the Standards Division of the Spine Intervention Society (SIS), where the authors looked at the effectiveness and risks of fluoroscopically guided parallel (also called conventional) RFN of the CMBN [5].

More recently, multi-tined trident cannulae have been developed that allow for end-on lesioning of the medial branch nerves. In a recent ex vivo study, multi-tined trident cannulae with a distal deployment mechanism demonstrated stable lesion characteristics at varying approach angles to the periosteal plane, while conventional sharp curved and 2-tined sharp straight cannulae did not [6]. The authors demonstrated that the trident cannulae created a pyramidal lesion closest to the tip that could create a stable lesion size up to an angle of 90° to the periosteal surface. Later, in a retrospective study, the authors reported that RFN of the CMBN with multi-tined trident cannulae conferred greater pain relief at 2 months post-procedure, as well as shorter procedure and fluoroscopy times when compared to lesioning with sharp straight conventional and 2-tined sharp straight cannulae [7].

### Rationale

The ex vivo study by Finlayson et al. [6] and the clinical retrospective study [7] provided pre-clinical and clinical evidence, respectively, that multi-tined trident cannulae may provide more consistent clinical results when utilized in the setting of RFN of the CMBN in patients with neck pain secondary to cervical facet joint disease. However, high-quality clinical evidence from an adequately powered study is required to evaluate the clinical outcomes of RFN of the CMBN utilizing an end-on approach with multi-tined trident cannulae, as compared to utilizing a parallel approach with a conventional sharp straight needle (the recommended SIS technique). The proposed *End*-on Versus *Pa*rallel *R*adiofrequency *L*esioning for Neurotomy of the Cervical Medial Branch Nerves (the EndPaRL study) will be the first prospective, randomized, outcome assessor-blinded, clinical trial comparing the clinical efficacy of sharp straight conventional versus multi-tined trident cannulae for RFN of the CMBN in patients with chronic neck pain due to cervical zygapophyseal joint osteoarthritis. The results of this study will help understand the role, benefits, and adverse effects of end-on lesioning techniques for RFN of the CMBN in the context of moderate-to-severe cervical zygapophyseal joint osteoarthritis while comparing this technique to the conventional parallel RFN approach.

### Objectives

The aim of our trial is to compare the efficacy of the recommended SIS technique utilizing a conventional parallel approach against the end-on approach for RFN of the CMBN in patients presenting with chronic, moderate-to-severe, neck pain in the context of cervical facet joint disease. The following are the primary objectives of this trial:

1. To compare the difference in mean Numerical Rating Scale (NRS) for pain scores at 3 months after the study interventions.
2. To compare the proportion of patients with a positive analgesic response (defined as 50% or greater reduction in the NRS score for neck pain as compared to baseline) at 3 months after the study interventions.

The following are the secondary objectives:

1. To compare the difference in mean NRS for pain scores at 1, 6, and 12 months after the study interventions.
2. To compare the proportion of patients with a positive analgesic response at 1, 6, and 12 months after the study interventions.
3. To compare the proportion of patients with 10 points or greater reduction in the Neck Disability Index (NDI) score (the minimal clinically important difference (MCID) [8]) at 1, 3, 6, and 12 months after the study interventions.
4. To compare the proportion of participants reporting some or much improvement in the Patients’ Global Impression of Change (PGIC) scale at 1, 3, 6, and 12 months after the study interventions [9].
5. To compare the mean Pittsburgh Sleep Quality Index (PSQI) scores and EuroQol (EQ-5D-5L) scores at 1, 3, 6, and 12 months after the study interventions [10].
6. To compare the duration of performing the study interventions, patient discomfort, radiation dose, and cost of the study interventions.
7. To compare average physical activity and sleep duration as measured by wrist-worn actigraphy over 1 week before the procedure and 1-month follow-up after the study interventions.
8. To assess the incidence of peri-procedural complications and post-procedural adverse effects during and at 1, 3, 6, and 12 months after the study interventions.
9. To assess opioid requirements in daily oral morphine equivalents in milligrams averaged over the 1 week prior to the 1-, 3-, 6-, and 12-month follow-ups after the study interventions.
10. To assess the patient assumption of the group they were assigned to at 3-month follow-up after the study interventions.

### Hypotheses

The primary hypotheses for this non-inferiority trial are that end-on lesioning for RFN of the CMBN is non-inferior to the parallel conventional technique for its impact on the intensity of pain relief and the proportion of patients with a positive analgesic response at 3 months after the intervention in patients with chronic, moderate-to-severe neck pain in the context of cervical zygapophyseal joint syndrome. The secondary hypotheses are that end-on lesioning for RFN of the CMBN is non-inferior to the parallel conventional technique for its impact function, quality of life, sleep, adverse effects of the interventions, analgesic intake at specified time points within 1 to 12 months after the interventions in patients with chronic, moderate-to-severe neck pain in the context of cervical zygapophyseal joint syndrome.

### Trial design

The EndParl study is designed as a prospective, multicentre, active comparator-controlled, non-inferiority randomized trial with two parallel groups and blinding of participants and outcome assessors. Randomization will occur on the day of the procedure and will be done to one of two arms on a 1:1 allocation basis. The randomization sequence will be computer-generated, and allocation concealment will be ensured by sequentially numbered, sealed opaque envelopes. This process will be carried out by an independent research coordinator, who will not be involved in the recruitment process or conduct of the trial. An unblinded physician will do the procedure but they will not be involved in assessing the outcomes. A blinded member of the research team will perform follow-up outcome assessments. Treating physicians, participants, close contacts, trial coordinators, and primary outcome analysts will be blinded to treatment allocation.

## Methods

### Trial setting

The study will be conducted at the Pain Medicine Clinics affiliated with the Division of Pain Medicine at the University of Toronto (Toronto, Ontario, Canada), and at the Comprehensive Integrated Pain Program (Toronto Western Hospital, Toronto).

### Eligibility criteria

**Fig. 1:**
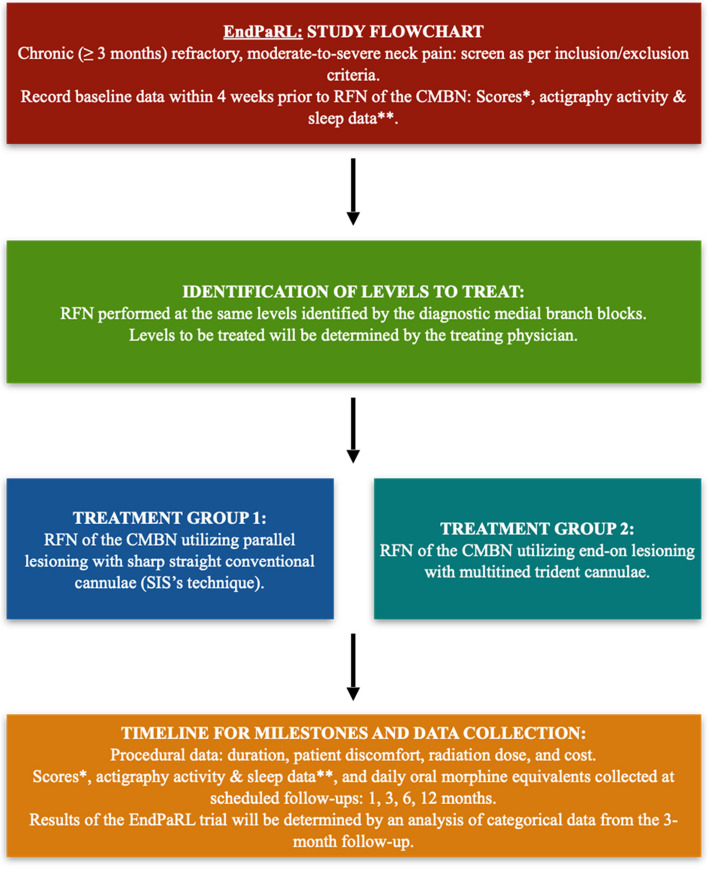
Study flow chart

The inclusion and exclusion criteria are summarized in Table 1. A flow chart of the trial is provided in Fig. 1.

**Table 1:** Inclusion and exclusion criteria

**Inclusion criteria**
1. Age 18 to 85 years
2. Predominant axial (non-radicular) neck pain for at least 3 months
3. 7-day average NRS score for neck pain of 5/10 or greater at baseline evaluation
4. Moderate or greater functional impairment due to pain, defined as NDI Questionnaire raw score of 15 out of 50 (30%) or greater
5. Failure to respond to conservative medical management (pharmacologic, physical therapy) for at least 3 months. 6. Positive response to two consecutive diagnostic blocks of the CMBN with a short and long-acting anesthetic, respectively, defined as reporting at least 80% relief of index pain concordant with the expected duration of the local anesthetics
**Exclusion criteria**
1. Participants with financial incentives or litigation associated with ongoing pain
2. Inability to complete assessment study-related outcome instruments
4. Widespread pain in the body
5. Prior RFN of the CMBN within the last 6 months at the time of enrollment
5. Severe mental health issues
6. Pregnancy or other reasons that preclude the use of fluoroscopy
7. Untreated coagulopathy
8. Systemic or local infection at the time of screening

### Interventions

#### Interventions common to both arms

Pre-procedure management will be identical for participants in the two groups and will be conducted at our interventional suites at the study sites. This includes obtaining peripheral intravenous access and application of routine electrocardiogram, non-invasive blood pressure, and pulse oximeter monitors. Oxygen by nasal prongs or mask and light sedation with midazolam and or fentanyl at the Anesthesiologist’s discretion will be provided. The RFN procedure will be performed at the same levels as identified by the diagnostic medial branch nerve blocks.

#### Study intervention and active comparator groups

*Study intervention*: Radiofrequency neurotomy of cervical medial branch nerves (CMBN) with end-on lesioning with multi-tined trident cannulae will be performed.

*Active comparator*: RFN of CMBN with parallel lesioning with sharp straight conventional cannulae (IPSIS’s technique)

The levels selected for diagnostic procedures will be determined by the treating physician based on the overall clinical picture including the location of pain, pain referral patterns, and imaging findings. The RFN parameters will be identical for the two active treatments (82° Celsius +/− 3–5° to accommodate the fluctuations that occur during ablation for 90 s), except for cannulae placement. The technique described in the SIS Practice Guidelines will be used for parallel lesioning cannulae placement [11]. For end-on placement of the multi-tined cannulae (Trident^®^, Diros Technology Inc, Markham, Ontario, Canada), the patient will be in the lateral position and the target will be the joint space between inferior articular process of C2 and superior articular process of C3 for the third occipital nerve, the middle of the facet pillars for the third to fifth cervical levels, and the superior part of the sixth and seventh cervical facets.

#### Post-procedural management

Following completion of the procedure, participants will be taken to the post-procedure recovery unit. Any complications related to the procedure will be recorded and managed if required. Participants will be discharged from the unit as per routine hospital policy. As per standard of care, participants will be advised to take one to two tablets of Percocet (containing 5 mg of oxycodone and 325 mg of acetaminophen) or Tylenol number 3 (containing 30 mg of codeine and 325 mg of acetaminophen) every 6 h if required to a maximum of 8 tablets in 24 h if they have pain intensity NRS scores higher than 3 out of 10. Frequency of use of these medications by study participants will be recorded and the average daily opioid consumption in oral morphine equivalents for the 1 week preceding the follow-ups at 1, 3, 6, and 12 months will be recorded.

In the event a subject is randomized but does not receive the trial interventions, the treatment will not be reassigned. Trial personnel will assess the patient and collect data throughout their enrollment in the trial. Participants in both arms will wear an actigraphy device (Appendix 1) to assess the quality and duration of sleep and activity for 1 week prior to the trial treatments (for collection of baseline data) and starting on the day of intervention for 1 month after to longitudinally assess the impact of the trial treatments on sleep and physical activity.

#### Modifications

The study participants have the right to withdraw from the study at any time. Any withdrawals will be documented along with the reason provided by the participant. If the reason for removal of a subject from the study is an adverse effect, the principal specific event will be recorded in the case report form. The subject will be followed until the adverse effect is resolved, if agreed by the subject.

#### Concomitant care

There will be no restrictions or changes to the baseline home medications, but study participants will be advised to avoid introduction of new analgesic agents in the post-treatment period.

#### Rescue medication and risk management

Any other pain medications provided during the study period will be considered rescue therapy. Participants will be given symptomatic treatment, and standard care will be provided. If any side effects or complications arise due to the study interventions, emergency unblinding will only occur when knowledge of the intervention is essential for medical care as determined by the Principal Investigator. In the event of emergency unblinding of one or more research team members, the timing, reason, and personnel involved will be recorded in the case record form (CRF), and blinding will be maintained for as many other trial personnel as possible.

### Outcomes

Patients will be assessed at baseline and 1, 3, 6, and 12 months after the study interventions. The assessment schedule and parameters are summarized in Table 2.

**Table 2:** EndPaRL assessment schedule

**Outcome**	**Measure**	**Baseline**	**Immediate after procedure**	**1 month after procedure**	**3 months after procedure**	**6 months after procedure**	**12 months after procedure**
**Patient demographics**		**X**					
**Pain intensity**	NRS	**X**	**X**	**X**	**X**	**X**	**X**
**Neck disability**	NDI	**X**	**X**	**X**	**X**	**X**	**X**
**Global improvement**	PGIC			**X**	**X**	**X**	**X**
**Sleep quality**	PSQI	**X**		**X**	**X**	**X**	**X**
**Sleep duration**	In hours and minutes from actigraphy device	**X**		**X**			
**Physical functioning**	Time spent at different activity levels from actigraphy device^b^	**X**		**X**			
**Opioid intake**	Daily OME in mg	**X**		**X**	**X**	**X**	**X**
**Analgesics**	Name and dose	**X**		**X**	**X**	**X**	**X**
**Procedure** **(1) Duration** **(2) Cost** **(3) Discomfort** **(4) Radiation**	(1) In minute (2) In CAD (3) 0–10 (4) Dose in mSv		**X** **X** **X** **X**				
**Complications**	e.g., bleeding, hematoma		**X**				
**Adverse effects**				**X**	**X**	**X**	**X**
**Health-related quality of life**	EQ-5D-5L^a^	**X**		**X**	**X**	**X**	**X**

*CAD* Canadian dollars, *NRS* Numerical Rating Scale, *OME* Oral morphine equivalents

^a^EuroQol 5-level EQ-5D version (EQ-5D-5L); Neck Disability Index (NDI); Numerical Rating Scale (NRS); Patients’ Global Impression of Change (PGIC); Pittsburgh Sleep Quality Index (PSQI)

^b^Data on physical activity and sleep collected using participant-worn actigraphy devices (light/moderate/severe)

The following are the *primary outcomes*:

1. The between-group difference in mean NRS for pain intensity scores at 3 months after the interventions.
2. The between-group difference in the proportion of patients with a positive analgesic response (defined as 50% or greater reduction in the NRS score for neck pain as compared to baseline) at 3 months after the interventions.

The following are the *secondary outcomes*:

1. Pain intensity: The between-group difference in mean NRS for pain intensity scores at 1, 6, and 12 months after the interventions.
2. Incidence of analgesic response: The between-group difference in the proportion of patients with a positive analgesic response (defined as 50% or greater reduction in the NRS score for neck pain as compared to baseline) at 1, 6, and 12 months after the interventions.
3. Neck pain-related disability: The between-group difference in the proportion of patients with a 10% or greater reduction in the Neck Disability Index (NDI) score at 1, 3, 6, and 12 months after the interventions.
4. Participant-assessed improvement: The between-group difference in the proportion of participants reporting some or much improvement on the Patients’ Global Impression of Change (PGIC) scale at 1, 3, 6, and 12 months after the interventions.
5. Quality of sleep and life: The between-group difference in the mean Pittsburgh Sleep Quality Index (PSQI) scores and EuroQol (EQ-5D-5L) scores at 1, 3, 6, and 12 months after the interventions. Sleep health will be assessed using PSQI, and actigraphy-measured variables respectively, including sleep duration, sleep quality, daytime sleepiness, sleep timing, and wakefulness after sleep onset.
6. Procedure-related outcomes: The between-group difference in the duration of procedure, discomfort, radiation dose, and cost of the procedures.
7. Actigraphy-collected physical activity and sleep: The between-group difference in the average physical activity and sleep duration as measured by wrist-worn actigraphy over 1 week prior to the procedure and 1 week prior to the 1-month follow-up after the interventions.
8. Adverse effects of the interventions: The between-group difference in the incidence of peri-procedural complications and post-procedural adverse effects at 1, 3, 6, and 12 months after the interventions.
9. Analgesic requirements: The between-group difference in opioid requirements as measured by daily oral morphine equivalents in milligrams averaged over the 1 week prior to the 1, 3, 6, and 12 months follow-ups after the interventions.
10. The patient assumption of the group they were assigned to at 3-month follow-up after the study interventions

### Participant timeline

Participants who meet the inclusion criteria will be reached by research coordinator for possible enrollment. Participants will be enrolled in the study after consenting and before receiving a RFN of the CMBN. The goal of this trial is to demonstrate non-inferiority of the end-on technique as compared to the conventional parallel technique for RFN of the CMBN. The baseline study questionnaires will be completed within 4 weeks before the study intervention. Baseline data on sleep and physical activity will also be collected using actigraphy over 1 week prior to the trial intervention and for at least 1 week prior to the follow-up at 1 month after the intervention. Routine scheduled study follow-ups will occur at 1, 3, 6, and 12 months after the study interventions, at which times all follow-up measures will be obtained (Table 2, Fig. 1). The follow-up at 1 and 3 months will be in-person whereas the follow-ups at 6 and 12 months may be virtual (video/phone) to assess trial-relevant outcomes.

### Sample size

The sample size was calculated using the Statistical Package for the Social Sciences (SPSS, IBM SPSS Statistics) software. A sample size of 72 participants (36 in each treatment group) will provide 80% power to detect a difference (inferiority margin), at 3 months after the intervention, in the mean NRS pain intensity scores (0–10 scale) between the two treatment groups being less than or equal to 1 point, given a standard deviation (SD) of 1.7 at the 0.05 significance level (alpha) using a two-sided two independent sample *t*-test. To satisfy both the non-inferiority and superiority hypothesis, we plan to recruit a total of 80 participants to account for a presumed 10% dropout rate. The sample size calculations and the assumptions underlying it are based on data published on RFN of nerves supplying other axial joints (lumbar facets, sacroiliac) for the relief of spinal pain [12, 13]. The chosen non-inferiority margin of less than or equal to 1 point (10%) is reasonable because a minimal clinically important difference in pain scores for a treatment to be considered efficacious is 2 points or 30% [14]. A non-inferiority study is justified to compare the end-on and parallel RFN lesions because similar efficacy is expected but there may be a lower incidence of adverse effects (e.g., patient discomfort) and better resource utilization (e.g., faster procedure time, lower radiation dose) with the end-on RFN technique.

### Recruitment

#### Feasibility of recruitment

Participants will be recruited from the Comprehensive Integrated Pain Program – Interventional Pain Service (CIPP-IPS) clinic at Toronto Western Hospital and the Pain Medicine Clinics affiliated with the Division of Pain Medicine at the University of Toronto. Around 15 patients undergo RFN of the CMBN every month at the study sites. Assuming a study enrollment rate of 50%, we should be able to recruit 7 to 8 participants every month. We expect to enroll the required sample size for the study over 9 to 12 months. There are two other academic pain centers and two community pain clinics in the Toronto area that may be approached to become study enrollment sites if the proposed enrollment is lower than anticipated.

#### Recruitment strategy

The Principal Investigator will introduce the trial to physicians and surgeons who refer patients with neck pain secondary to cervical facet joint syndrome the study sites. The referring physicians and surgeons will be encouraged to refer patients with chronic neck pain who meet the inclusion criteria.

### Assignment of interventions

#### Randomization, sequence generation, and allocation concealment

Upon participant enrollment, patients will be randomized to one of two arms, with a 1:1 allocation. The randomization sequence will be computer-generated by a member of the research team. An independent research coordinator will handle the treatment allocation process. This coordinator will ensure allocation concealment by using sequentially numbered, sealed opaque envelopes. The allocation sequence will be provided to an attending staff pain medicine physician.

### Blinding

#### Blinding mechanism

Participants, physicians, and all outcome assessors will all be blinded during the conduct of the trial and statistical analysis. Assessors will remain blinded to their group assignments throughout the study. A Staff Pain Medicine physician with at least 5 years of experience in performing RFN of the CMBN and who is not involved in the assessment of outcomes will receive the randomization assignment and perform the procedure using the assigned technique. Biostatisticians who will analyze the data will be blinded to group allocation and the two study groups will be represented as I and II on the study database with no other identifiers.

#### Emergency unblinding

Emergency unblinding will only occur when knowledge of the treatment assignment is necessary for the immediate medical management of a participant. This can occur in the occurrence of serious adverse events, or by decision of the Principal Investigator or the Data Safety Monitoring Board. Time and reason for emergency unblinding will be recorded. In the event of emergency unblinding, the timing, reason, and personnel involved will be recorded in the CRF, and blinding will be maintained in as many other trial personnel as possible.

### Data collection

#### Data collection methods

Data will be gathered using data collection forms, and the research coordinator will input the information into a password-protected electronic database (Microsoft Excel platform). Our data collectors are experienced research assistants who have been trained in Good Clinical Practice (GCP). There will be regular meetings between the data collectors and the trial Principal Investigator. We will use validated questionnaires for assessing pain and related domains. Patients will be followed closely at dedicated time points to promote retention and collection of follow-up data.

#### Baseline data

Baseline data will include participants demographics, pain intensity as measured by NRS score, physical activity, sleep duration both measured by Actigraphy, sleep quality measured by PSQI, physical function as measured by the NDI, opioid and non-opioid analgesic intake.

#### Intervention data

Duration of the procedure will be recorded as well as cost, discomfort, dose of radiation, and any intra- and immediate post-procedural complications.

#### Follow-up data

The following data will be collected at 1, 3, 6, and 12 months following the interventions. The data points will include pain intensity as measured by NRS score, participant impression of improvement as measured by PGIC, opioid and non-opioid analgesic intake, physical activity as measured by the NDI, health-related quality of life measured by EQ-5D-5L, physical activity and sleep both measured by Actigraphy, and adverse effects.

#### Data management and confidentiality

All trial data will be checked on a regular basis for errors and missing data. Trial data from the actigraphy device will be exported as a comma-separated values (CSV) file for analysis. It will be locally synced on an institutional research laptop. Participants’ contact information and identifying information will be encrypted and stored in a separate database. Identifying information will be deleted once follow-up is completed. The Institutional Research Ethics Board (IREB) audits trial records and collected personal health information to verify that the information collected for the trial is correct and to make sure the trial is following institutional regulations. Completed questionnaires collected during the trial visits will be labeled with a unique trial code. No patient names will be used. The information that links the unique trial code to the participant will be kept in a secure area in our institute, and it will be distinct from the participants’ trial data. This information can only be accessed by research team members who are involved in the trial. All records and documents pertaining to the trial will be retained by the trial sites for 5 years from the completion of the trial.

In the event of personal health information disclosure to an unauthorized party, the following will be done: any further release of information will be stopped, as much information as possible will be retrieved, IREB privacy Offices will be contacted, and further actions would be taken based on their recommendations.

### Statistical methods

A statistician will be consulted to assist in data analysis and interpretation. Descriptive information for the sample’s study measures will include frequency distributions or proportions for categorical variables and means (standard deviations) or medians (interquartile ranges) (if non-normally distributed) for the continuous variables. To compare data between the two groups, categorical outcomes will be analyzed with the chi-square test and continuous variables will be analyzed with independent *t*-tests (or Wilcoxon rank sum test, as appropriate). A two-sided *p*-value of < 0.05 will be considered statistically significant. Both intention-to-treat (based on study arm allocation) and per-protocol (based on the participants who adhered to the study protocol and provided data at study outcome time points) analysis will be performed and reported.

Correlation between reduction in pain and improvement in function will be evaluated with a longitudinal model of changes from baseline to 3-month, 6-month, and 12-month follow-ups. An intention-to-treat strategy will be used for all analyses. Differences in treatment effects and 95% confidence intervals for pain and secondary outcome scores will be calculated using chi-square and odds ratios for dichotomous variables, and ANOVA (with *t*-tests for pairwise comparisons) and Mann–Whitney for continuous variables, as indicated. A set of logistic regression models for categorical outcome at 3 months will be created using variables hypothesized to have an effect on treatment (gender, intensity of pain, nature of injury), as well as those found to have a *p*-value less than 0.20 in univariate analysis. A secondary analyses based on data from participants who undergo unilateral versus bilateral RFN with each of the two study treatments is also planned. Missing data will be imputed using the “last observation carried forward” approach if at least 80% of the outcome data is available for the participant. All analyses will be performed using the SPSS program.

### Data monitoring

#### Formal committee

An independent Data Safety Monitoring Board (DSMB) whose members have experience in clinical trials has been formed. Monitoring and reporting of adverse events will be conducted throughout the whole trial. If there are concerns about the quality of care, and or the safety of any interventions, the committee will have the authority to stop the trial subject to evaluation by the Institutional REB.

#### Study coordinating center and study committees

The Principal Investigator (PI) and the Study Coordinator (SC) will be based at Toronto Western Hospital, Toronto, Canada. This will be the coordinating center for the study. The Co-Investigators of this Study work at the various study sites. The PI and the SC will be responsible for preparation of the protocol and any revisions, preparation of study data collection forms (case report forms), and drafting the research consent forms.

The Steering Committee consists of the PI (AA), SC (DA), biostatistician (XC), and the senior investigator (AB). The Steering Committee will meet once every 6 months and its role will be to approve the main study protocol and any amendments, monitor and supervise the trial towards its overall objectives, review relevant information from other sources, and resolve any problems that arise at a system level. The Trial Management Committee consists of the PI (AA), SC (DA), two co-investigators (ZM, JK), and the senior investigator (AB). The role of this Committee will be to monitor all aspects of the conduct and progress of the trial, ensure that the protocol and the budget are adhered to, and take appropriate action to safeguard participants and the quality of the trial itself. The Data Monitoring Committee consists of clinicians and biostatisticians appointed by study sponsors who will provide independent assessment of the safety, scientific validity, and integrity of the study.

### Interim analysis

There will be no planned interim analysis unless any safety concerns arise during the conduct of the trial.

### Safety/harms

Continuous monitoring and documentation of any adverse events will be carried out throughout the trial. Any unexpected adverse effects will be recorded in the participant’s file and reported to the Institutional REB. Adverse events will be collected non-systematically (i.e., spontaneous reporting from open-ended questions during follow-ups) but these will be classified as non-serious (e.g., pruritus in the area of the RFN) and serious (e.g., neurological deficits) for the purpose of reporting of the study’s results.

#### Auditing

Formal audits will be conducted at the request of DSMB or Institutional REB. Representatives from the Institutional Review Board/Independent Ethics Committee can examine trial records and personal health information to verify the accuracy of collected data. The sponsor has the responsibility to ensure that investigators and institutions involved in the trial allow monitoring, audits, Institutional Review Board/Independent Ethics Committee (IEC) reviews, and regulatory inspections. This includes providing direct access to source data/documents for these purposes.

### Ethics and dissemination

#### Research ethics approval

The EndPaRL study will be conducted in accordance with the ethical principles laid down in the Declaration of Helsinki, the protocol, Good Clinical Practice guidelines, and applicable regulatory requirements. Full written informed consent will be obtained prior to conducting any study activities. The study was reviewed and approved by the Research Ethics Board (REB). This trial has been registered on ClinicalTrials.gov website (NCT05818774), and results will be reported as per the Consolidated Standards of Reporting Trials (CONSORT) guidelines. Protocol modifications will be avoided but, if necessary, will be communicated to all relevant stakeholders including Institutional REB committees, participants, trial registry, and regulators.

### Criteria for subject withdrawal

Subjects have the right to withdraw from the study at any time, without providing a reason. In the event that a subject decides to prematurely discontinue the study, he/she will be asked if he/she can still be contacted for further information. This will be documented accordingly. If the subject has already received the study drug prior to withdrawal, the subject will be requested to be contacted for a safety follow-up visit.

When applicable, subjects should be informed of circumstances under which their participation may be terminated by the Investigator without their consent. The Investigator may withdraw subjects from the study in the event of intercurrent illness, adverse events, lack of compliance with the study or study procedures, any other reasons where the Investigator feels it is in the best interest of the subject, or at the request of the Research Ethics Board (REB). The reasons for withdrawal by the Investigator will be documented and explained to the subject. Should a subject decide to withdraw, all efforts should be made to complete and report the observations, particularly the follow-up questionnaires (if agreed to by the subject), as thoroughly as possible. If the reason for the removal of a subject from the study is an adverse event, the principal specific event will be recorded in the case report form. The subject will be followed until the adverse event is resolved if agreed by the subject.

### Ancillary and post-trial care

No additional provisions will be made for post-trial care, and routine clinical care will be provided by the participant’s primary physician. If patient harmed as a direct result of taking part in this study, all necessary medical treatment will be made available to no cost for patient.

### Dissemination policy

Study results will first be disseminated at local, national, and international conferences and submitted for publication in a peer-reviewed journal. Authorship on the manuscript submitted for publication will be based on the contributions of the authors to the study.

### Additional studies

If the EndPaRL study achieves non-inferiority for end-on radiofrequency lesioning, we plan to perform a cost-effectiveness study to compare the two techniques for RFN of the CMBN in patients presenting with chronic, moderate-to-severe neck pain in the context of cervical zygapophyseal joint osteoarthritis.

## Discussion

Pain in the neck has a high prevalence and the cervical facet joints affected by trauma or degeneration are often generators of this pain. There are limited effective options for treating this pain with only RFN showing evidence of effectively reducing pain from cervical facet joint dysfunction [9]. The purpose of RFN is to ablate the CMBN using high temperatures and provide longer pain relief than simple nerve or intra-articular blocks. The procedure involves first positioning a needle electrode over the nerve at its corresponding bony anatomy. The conventional technique for performing RFN of the CMBN involves introduction and advancement of the RF cannula from a posterior to anterior direction that is parallel to the cervical facets and the CMBN that run on these facets. The efficacy of this approach has been demonstrated in several studies [2–5]. More recently, an end-on technique has been proposed that involves introduction and advancement of the RF cannula from a lateral to medial direction that is perpendicular (end-on) to the cervical facets and the CMBN, thereby reducing the cannula path length and discomfort for the patient. However, the efficacy, benefits, and adverse effects of this end-on approach have not been compared to the conventional parallel approach.

This non-inferiority study aims to compare the efficacy and effectiveness of the conventional parallel approach versus the end-on approach for RFN of the CMBN in patients with cervical facet joint syndrome. Eighty participants will be included and will be divided between the two groups. Participants and assessors will be blinded to the treatment allocation. Data on will be collected from baseline up to 12 months after the RF procedure on the analgesic benefit, neck-related disability, physical activity, sleep, participants’ impression of change, procedural complications, adverse effects, and analgesic requirements. The methodology will involve the use of wearable technology to assess physical activity and sleep, validated questionnaires for all the outcome domains, and it will be the first to compare the two techniques of RFN of the cMBN.

This trial will help to identify if the end-on approach for RFN of the CMBN is an effective and safe alternative to the conventional parallel technique for relieving neck pain originating from the cervical facet joints. The investigators hope the knowledge from this trial will be widely implemented to improve patients’ health and quality of life.

## Trial status

Protocol version and date: version 3.0, July 27, 2023

Recruitment initiated from March 10, 2023

Estimated end of recruitment: November 2024

Estimated trial completion date: March 2025

### Supplementary Information

**Additional file 1:** **Appendix 2.** Participation consent form.

## Data Availability

Following the completion of data entry, only the trial investigators and trial coordinators will have access to the final trial dataset.

## Appendix 1

### Actigraphy monitoring

This is a non-invasive method to assess sleep and activity. An accelerometer-based wrist-worn device (GENEActiv®, Activeinsights, Cambridge, UK) records activity, light and temperature continuously for up to one month. The unprocessed , accessible in an open data format, can be analyzed using specific algorithms.

## Abbreviations

CMBN: Cervical medial branch nerves
CONSORT: Consolidated Standards of Reporting Trials
EQ5D-5L: EuroQol 5-level EQ - 5D version - 5 levels
NDI: Neck Disability Index
NRS: Numerical Rating scale
OME: Oral Morphine equivalents
PGIC: Patients’ Global Impression of Change
PSQI: Pittsburgh Sleep Quality Index
REB: Research Ethics Board
RFN: Radiofrequency Neurotomy
SIS: Spine Intervention Society
