# Supplementary material for: End-on versus parallel radiofrequency lesioning for neurotomy of the cervical medial branch nerves: a study protocol of a prospective, randomized, double-blind clinical trial: the “EndPaRL” study
Source: Trials. 2023 Nov 11;24:721. doi: 10.1186/s13063-023-07752-9 (PMC10638800; doi:10.1186/s13063-023-07752-9)
Supplement: Supplementary file 1 — Additional file 1: Appendix 2. Participation consent form. [file 13063_2023_7752_MOESM1_ESM.docx]

**Appendix 2.** Participation consent form

**Consent Form for Participation in a Research Study**

**Title: Prospective, Randomized, Double Blind Clinical Trial Comparing End-on Versus Parallel Radiofrequency Lesioning for Neurotomy of the Cervical Medial Branch Nerves: The EndPaRL Study**

**Principal Investigator:** Anuj Bhatia, MBBS MD PhD FRCA FRCPC FFPMRCA

**Contact information:** T: (416) 603-5118

***24 HOUR EMERGENCY CONTACT***

Department of Anesthesia

Toronto Western Hospital

Pager: (416) 790-4448

# Introduction

You are being asked to take part in a research study. Please read this information about the study presented in this form. The form includes details on the study’s risks and benefits that you should know before you decide if you would like to take part. You should take as much time as you need to make your decision. You should ask the study doctor or study staff to explain anything that you do not understand and make sure that all of your questions have been answered before signing this consent form. Before you make your decision, feel free to talk about this study with anyone you wish including your friends, family, and family doctor. Participation in this study is voluntary.

**Background and Purpose**

You are being asked to take part in this research study because you are suffering from neck pain for more than 12 months and your doctor has recommended for you to undergo Radiofrequency neurotomy (RFN).

Radiofrequency neurotomy (RFN), also called radiofrequency ablation (RFA) is a technique where a needle is used to deliver heat to a very precise area of the nerves (known as ablation) under fluoroscopy (x-ray) guidance. By heating or ablating the nerve, the transmission of pain signals is interrupted, that may result in pain relief in that area. Using RFN/RFA to relieve pain is considered the standard of care at UHN for various pain areas. .

There are currently two techniques on how Radiofrequency neurotomy (RFN) is delivered:

1. Inserting the needle parallel to the nerve with a ‘sharp-straight’ cannula
2. Inserting the needle directly on the nerve with a multi-tined (or trident) cannula

The first technique is considered the standard method of treating chronic neck pain in Ontario. More recently, the second technique has been adopted by other centers (including UHN) as it has the potential for creating a more stable lesion size (burning site) for the procedure and for more accurate targeting of the nerves. This study is being done to directly compare these two techniques, and find out which technique is the most effective and most satisfactory for patients with neck pain. Approximately 72 patients will take part in this study at Toronto Western Hospital.

# Study Design

This is a randomized double blinded study. If you decide to participate you will be "randomized" into one of the study groups described below. Randomization means that you are put into a group by chance. It is like flipping a coin. Neither you nor your doctor can choose what group you will be in. You will have a *50/50* chance of being placed in *either* group. Neither you nor your doctor will know which group you are in. In an emergency, if the study *intervention* needs to be identified, the doctor can get this information.

- ***Group 1*-**You will have the RFN procedure using a sharp straight cannula, where the needle is inserted parallel or alongside the nerve
- ***Group 2*- You** will have the RFN procedure using a multi-tined trident cannulas, where the needle is inserted directly on the nerve (*end-on technique)*


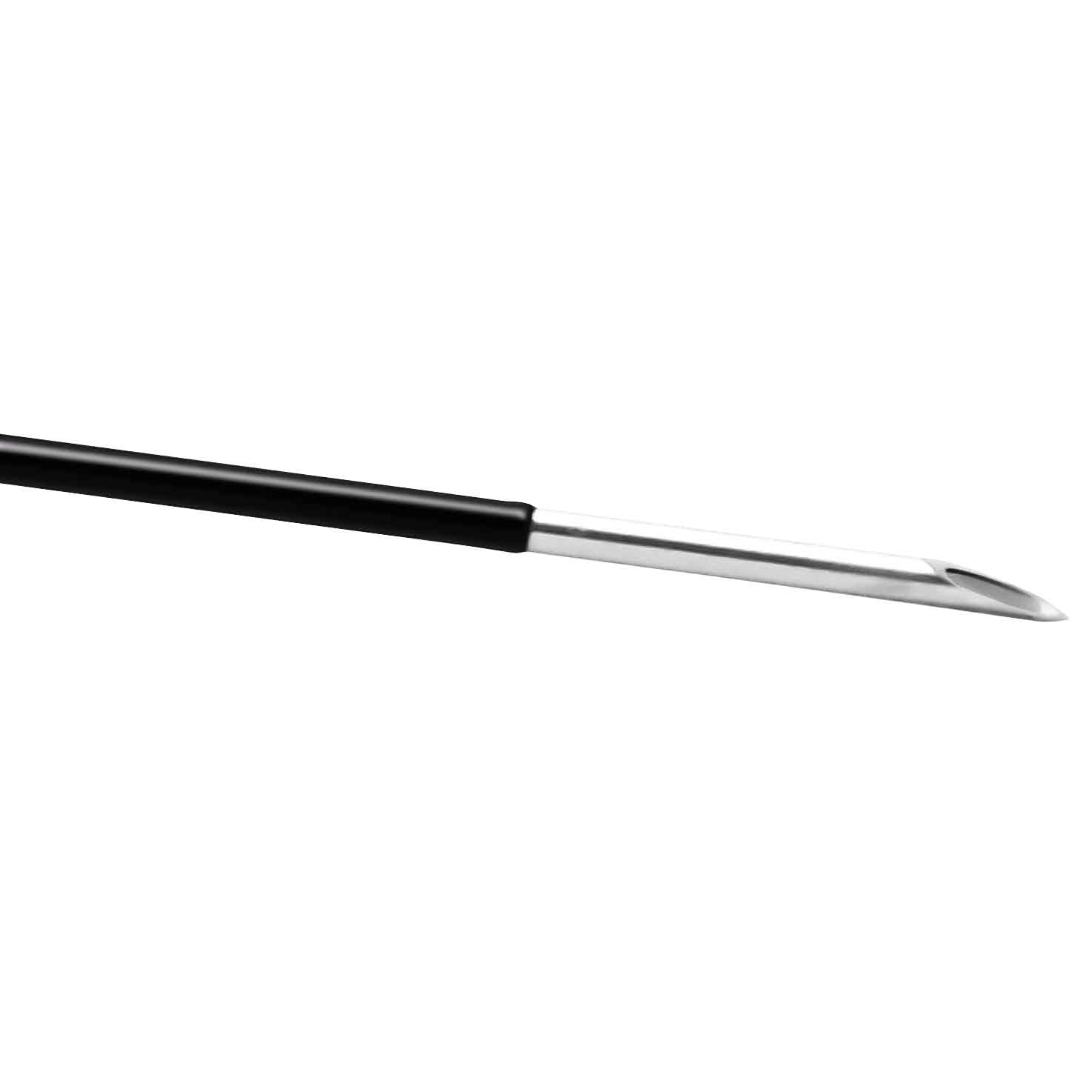

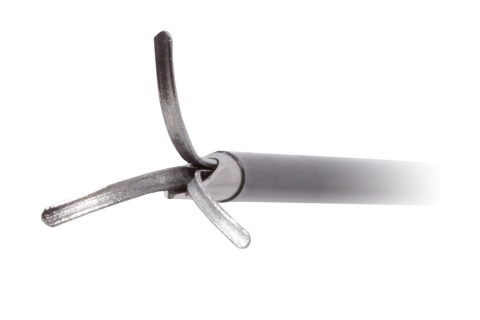

Figure 1. Cannula tips for Group 1 (left) and Group 2 (right)

The procedure will be similar for all the participants except for the type of the cannulas used.

We expect the total duration study to last for about 18 months, and your participation in the study to last about 3 months.

# Study Procedures

If you agree to be in this study, you will be asked to sign this consent form. Some interactions can be done remotely over phone or by email, except the procedure, which will be done at the hospital. An option for a video or telephone visit follow-up will be provided to you if you are not able to visit the clinic.

The study is divided into 3 study Periods:

- Screening Period
- RFN Procedure Period
- Follow up Period
- Screening Visit *(within 4 weeks before the procedure)*:

This visit may take up to 30-40 mins. You will undergo the following study procedures:

- - - Demographic Information collection: You will be asked about your age, gender, height and weight, employment status, smoking , alcohol and substance use etc. Information will also be collected from your medical charts or standard of care questionnaires completed in the clinic.
    - Medical History : You will be asked about your current health conditions, medications, and information about your neck pain
    - Complete Questionnaires: You will be asked to complete the following questionnaires on paper:
    - *Pain Intensity* score. This is a numeric rating scale (NRS) to rate your pain from 0-10
    - *Sleep quality*: Pittsburgh Sleep Quality Index (PSQI) to assess the quality, duration, and disruptionwith your sleep.
    - *Global improvement*: Patients' Global Impression of Change (PGIC), a self-reported measure reflecting patient's belief about the effectiveness of procedure
    - *Quality of life*: EuroQol-5 dimension (ED-5D-5L), to assess your overall quality of life
- Wear an Actigraph watch : You will be provided with an actigraph watch known as the GeneActiv., which will record your movements and sleep throughout the day The device will not collect your location information (GPS). You will be asked to wear this device for 4 weeks before your procedure. The device needs to be worn every day for 24 hours. This is waterproof and does not interfere with any body functions. You will be asked to bring the device on your procedure day. We will then provide you with another device after your procedure to wear again for next 4 weeks and compare the data before and after the procedure.
- RFN Procedure Period

Day of RFN procedure:

**Before the Procedure:**

- the following standard monitoring will be done:
  - ECG: An electrocardiogram is a test that measures the electrical activity of the heart. Patches attached by wires to a machine will be put on your chest so that the machine can record the pattern of your heart beats.
  - blood pressure measurement including oxygen levels via oximeter on your finger

**RFN Procedure:**

- A pain physician will perform the procedure. The level of the nerve ablation will be the same as identified by the diagnostic blocks you had previously.
- Light sedation will be provided through IV access. You will be awake at all times so the physicians can talk to you throughout the procedure.
- Fluoroscopy: This is a procedure where continuous X-ray image is shown on a monitor. The **RFN Procedure** will be performed via Fluoroscopy (X-ray guidance) In order to visualize the procedure

**After the Procedure:**

You will stay in the Recovery for approximately two hours from where you will be discharged.

Upon discharge, you will also receive the GENEActiv watch to wear again for next four weeks

As you will not be able to drive due to the drugs administered during the procedure, it is advised that you have someone to accompany you and arrange for transportation afterwards

- Follow ups *(1 month, 3 months, 6 months, and 12 months)*

After the procedure, you will be followed at 1 month, 3 months, 6 months, and 12 months. Each encounter will be expected to take about 15 minutes.

- Completion of questionnaires: A member of the research team will contact you to complete the questionnaires (these will include the same questions completed during the screening period). This can be done:
  - By phone, where the study coordinator will read the questions to you and record your responses
  - In person during your routine visits where you will be provided a paper copy of the questionnaires to complete.
  - By e-mail, where a copy of the questionnaires can be shared with you using our secure file sharing service. You may print the questionnaires to complete, and then scan and send these back to the study team.
- Sleep duration and Physical functioning *(GeneActiv watch*) – this will be recorded for a month, after which we will provide you with a shipping label and an associated envelope if needed to return the watch if you do not attend the 1-month follow-up appointment in-person
- Medication History: You will be asked about your opioid use and other pain medications
- Review of Side effects: You will be asked whether you experience any side effects

Please see the table below for a summary of what will be done at each study visit.

**Assessment schedule**

|  | **Screening** | **Procedure** |  | **Follow Up** | | | |
| --- | --- | --- | --- | --- | --- | --- | --- |
|  |  | Day of procedure | 1 Month | | 3 months | 6 Months | 12 Months |
| **Visit number**  (remote or in-hospital) | **1** | **2**  (in-hospital) | **3** | | **4** | **5** | **6** |
| **Screening and consent** | **X** |  |  | |  |  |  |
|  |  |  |  | |  |  |  |
| **Assessments** |  |  |  | |  |  |  |
| **Pain Intensity** (NRS) | **X** | **X** | **X** | | **X** | **X** | **X** |
|  |  |  |  | |  |  |  |
| **Questionnaires** | **X** |  | **X** | | **X** | **X** | **X** |
| **Daily opioid Intake** | **X** |  | **X** | | **X** | **X** | **X** |
| **Other pain medications** | **X** |  | **X** | | **X** | **X** | **X** |
| **Physical Functioning** (from actigraph watch) | **X** |  | **X** | |  |  |  |
| **ECG/Blood pressure** |  | X |  | |  |  |  |
| **Fluoroscopy** |  | **X** |  | |  |  |  |
| **Side effects** |  | X | **X** | | **X** | **X** | **X** |
|  |  |  |  | |  |  |  |
| Duration of Visit | **15 minutes** | **3 hours (1 hour procedure, 2 hours recovery)** | **15 minutes** | | **15 minutes** | **15 minutes** | **15 minutes** |

**Risks Related to Being in the Study**

Taking part in this study has risks. Some of these risks we know about. There is also a possibility of risks that we do not know about and have not been seen in humans to date. Please contact the

study doctor or study coordinator if you have any side effects even if you do not think it has anything to do with this study.

**Radiofrequency neurotomy (RFN), or Radiofrequency ablation (RFA):**

Please see the possible risks associated with the procedure below:

Less Common (*1 to 10%*): In 100 people undergoing the procedure, between 1 and 10 may have:

- - Superficial bleeding and bruising
  - Reduced or no pain relief
  - Increased pain at site of entry
  - Worsening of existing pain lasting several days
  - temporary sensory and motor loss (numbness and inability to move in the area of ablation)

Rare but Serious (less than 1%): In 100 people undergoing the procedure, fewer than 1 may have:

- - Risk of nerve injury (Long-term weakness and numbness)
  - Infection
  - Internal bleeding or bruising
  - Damage to blood vessels near the procedure area
  - Seizures,
  - Cardiovascular complications (irregular heartbeat during the procedure)
  - Skin burns.

# Risks of Sedation

Likely [Common] (*20 to 49%*) In 100 people receiving study drug, between 20 and 49 may have:

# Drowsiness during the day of the procedure

# Risks of local anesthetic:

Rare but Serious (less than 1%): In 100 people receiving the procedure, fewer than 1 may have:

- Seizures

# Risks of Fluoroscopy:

Rare but Serious (less than 1%): In 100 people receiving the procedure, fewer than 1 may have:

# Skin Burns

# Tumours

# Benefits

You may not receive benefit from being in this study. Information learned from this study may help in pain treatment of patients with similar pain conditions in future.

# Confidentiality

Your data will be shared as described in this consent form or as required by law. All personal information such as your name, address, phone number, OHIP number, and family physician’s name will be removed from the data and will be replaced with a number. A list linking the number with your name will be kept by the study doctor in a secure place, separate from your file.

Personal Health Information

If you agree to join this study, the study doctor and his/her study, team will look at your personal health information and collect only the information they need for the study. Personal health information is any information that could be used to identify you. It may include your:

- - Name
  - Age
  - Phone number
  - New or existing medical records that includes types, dates and results of medical tests or procedures

Your participation in this study will also be recorded in your medical record at this hospital for clinical safety purposes.

The following people may come to the hospital or be given remote access to an electronic portal (via the internet) to look at the study records and at your personal health information to check that the information collected for the study is correct and to make sure the study is following proper laws and guidelines. When using the electronic portal, we will share your medical record number using a secure method, so that your records are included as part of their review.

- Representatives of the University Health Network (UHN) including the UHN Research Ethics Board

These individuals have completed privacy training and signed confidentiality agreements and/or are required by law to keep your information confidential.

Whether on-site or remotely, UHN makes all efforts to ensure that your information is shared in a way that is secure and private (encrypted). However, any electronic communication carries some risk of third parties gaining unauthorized access to information.

The study doctor will keep any personal health information about you in a secure and confidential location for 10 years.

Research Information in Shared Clinical Records

If you participate in this study, information about you from this research project may be stored in your hospital file and in the UHN computer system. The UHN shares the patient information stored on its computers with other hospitals and health care providers in Ontario so they can access the information if it is needed for your clinical care. The study team can tell you what information about you will be stored electronically and may be shared outside of the UHN. If you have any concerns about this, or have any questions, please contact the UHN Privacy Office at 416-340-4800, x6937 (or by email at [privacy@uhn.ca](mailto:privacy@uhn.ca)).

Study Information that Does Not Identify You

You will not be named in any reports, publications, or presentations that may come from this study.

No personal information will be entered in the actigraph watch. The information that will be collected by the actigraph will be stored on a UHN computer for 10 years.

# Reminders

It is important to remember the following things during this study:

- - Ask your study team about anything that worries you.
  - Tell study staff anything about your health that has changed.
  - Tell your study team if you change your mind about being in this study.

# Voluntary Participation

Your participation in this study is voluntary. You may decide not to be in this study, or to be in the study now, and then change your mind later. You may leave the study at any time without affecting your care. You may refuse to **answer any question you do not want to answer, or not answer an interview question by saying, “pass”. We will give you new information that is learned during the study that might affect your decision to stay in the study**

# Alternatives to Being in the Study

You do not have to join this study to receive treatment for your condition. The following are approved medications/interventions for your condition:

- RFN procedure using a sharp straight cannula
- RFN procedure using a multi-tined trident cannulas
- Alternative pharmacological options including oral analgesics (pain medications) that can be reviewed by your pain physician.

# Withdrawal from Study

The investigator may decide to remove you from this study without your consent at any time for any of the following reasons:

- - The investigator decides that continuing in this study would be harmful to you.
  - You are unwilling to follow the study procedures.

If you decide to leave the study, you have the right to request withdrawal of information collected about you. Let your study doctor know.

No new information will be collected without your permission.

# Costs and Reimbursement

There is no extra cost involved in this study. You will be reimbursed $50 to cover travel and parking expenses for participating in this study.

# Rights as a Participant

If you are harmed as a direct result of taking part in this study, all necessary medical treatment will be made available to you at no cost.

By signing this form, you do not give up any of your legal rights against the investigators, sponsor-investigator or involved institutions for compensation, nor does this form relieve the investigators, sponsor or involved institutions of their legal and professional responsibilities.

# Conflict of Interest

All of the people in this study have an interest in completing this study. Their interests should not influence your decision to participate in this study. You should not feel pressured to join this study.

**Commercialization**

You will not receive any financial benefit that might come from the results of this study.

# Questions

If you have any questions, concerns or would like to speak to the study team for any reason, please call Dr. Anuj Bhatia at (416) 603-5118 or Study Coordinator at *416-603-5800 ext. 3959.*

If you have any questions about your rights as a research participant or have concerns about this study, please call the Chair of the University Health Network Research Ethics Board (REB) or the Research Ethics office number at 416-581-7849. The REB is a group of people who oversee the ethical conduct of research studies. These people are not part of the study team. Everything that you discuss will be kept confidential. You will be given a signed copy of this consent form. A description of this clinical trial will be available on [*http://www.ClinicalTrials.gov.*](http://www.ClinicalTrials.gov.) This website will not include information that can identify you. This will include a summary of the results.

# Consent

This study has been explained to me and any questions I had have been answered. I know that I may leave the study at any time. I agree to the use of my information as described in this form. I agree to take part in this study.

Study Participant’s Name Signature Date

My signature means that I have explained the study to the participant named above. I have answered all the questions.

Name of Person Obtaining Consent Signature Date

Was the participant assisted during the consent process?  **YES**  **NO**

If **YES**, please check the relevant box and complete the signature space below:

The person signing below acted as an interpreter, and attests that the study as set out in the consent form was accurately sight translated and/or interpreted, and that interpretation was provided on questions, responses and additional discussion arising from this process.

___________________

Print Name of Interpreter Signature Date

__________________________

Language

The consent form was read to the participant. The person signing below attests that the study as set out in this form was accurately explained to, and has had any questions answered.

___________________

Print Name of Witness Signature Date

Relationship to Participant
